# A Highly Sensitive and Selective Fluorescein-Based Cu^2+^ Probe and Its Bioimaging in Cell

**DOI:** 10.3389/fnut.2022.932826

**Published:** 2022-06-27

**Authors:** Xin Leng, Mengyao She, Xilang Jin, Jiao Chen, Xuehao Ma, Fulin Chen, Jianli Li, Bingqin Yang

**Affiliations:** ^1^Key Laboratory of Resource Biology and Biotechnology in Western China, Ministry of Education, Xi'an, China; ^2^Biomedicine Key Laboratory of Shaanxi Province, Xi'an, China; ^3^Lab of Tissue Engineering, Faculty of Life Science & Medicine, The College of Life Sciences, Northwest University, Xi'an, China; ^4^Key Laboratory of Synthetic and Natural Functional Molecule of the Ministry of Education, College of Chemistry & Materials Science, Northwest University, Xi'an, China; ^5^School of Materials and Chemical Engineering, Xi'an Technological University, Xi'an, China

**Keywords:** trace metal, fluorescent probe, copper ion, test strips, cells imaging

## Abstract

Copper is a vital trace metal in human body, which plays the significant roles in amounts of physiological and pathological processes. The application of copper-selective probe has attracted great interests from environmental tests to life process research, yet a few of sensitive Cu^2+^ tests based on on-site analysis have been reported. In this paper, a novel fluorescein-based fluorescent probe N4 was designed, synthesized, and characterized, which exhibited high selectivity and sensitivity to Cu^2+^ comparing with other metal ions in ethanol–water (1/1, v/v) solution. The probe N4 bonded with Cu^2+^ to facilitate the ring-opening, and an obvious new band at 525 nm in the fluorescence spectroscopy appeared, which could be used for naked-eye detection of Cu^2+^ within a broad pH range of 6–9. Meanwhile, a good linearity between the fluorescence intensity and the concentrations of Cu^2+^ ranged 0.1–1.5 eq. was observed, and the limit of detection of N4 to Cu^2+^ was calculated to be as low as 1.20 μm. In addition, the interaction mode between N4 and Cu^2+^ was found to be 1:1 by the Job's plot and mass experiment. Biological experiments showed that the probe N4 exhibited low biological toxicity and could be applied for Cu^2+^ imaging in living cells. The significant color shift associated with the production of the N4-Cu^2+^ complex at low micromolar concentrations under UV light endows N4 with a promising probe for field testing of trace Cu^2+^ ions.

## Introduction

Trace elements are present in living body in small amounts, but they are important for the growth, development, maintenance, and recovery of health (1–3). Either insufficient or excessive intake of trace elements could cause several diseases (4). Copper is a vital trace metal in the human body, which plays the significant roles in amounts of physiological and pathological processes including body circulation, ATP production, and bone formation as well as protecting the cell from oxygen free radicals (5–7). An aberrant concentration of copper may cause the imbalance in organisms, resulting in a series of pathological illnesses such as liver and kidney damage, cancer, and neurodegenerative disorders including Parkinson's, Wilson's, and Alzheimer's (8–10). In addition, Cu^2+^ pollution in water and soil mainly comes from the metal-containing wastes caused by industrial production. Due to the pollution of water environment and soil environment, Cu^2+^ can gradually be accumulated in animals and plants, thereby affecting human health (11, 12). Resulting ground the absence and overloading of Cu^2+^ has been found to adversely affect all the biological systems including humans (13–15). As a result, it is vital to develop efficient methods for tracking and quantifying the anomalous of the concentrations and distributions of Cu^2+^ to comprehend the transportation, metabolic mechanism, and interaction roles of Cu^2+^ in linked physiological and pathological processes (16–20).

In the past decades, many copper quantification methods including inductively coupled plasma mass spectrometry (ICP-MS) (21, 22), atomic absorption spectrometry (AAS) (23, 24), and fluorescent probes (25–27) have been reported. These methods offer sensitivity but usually suffer from complexity and costly. Fluorescent probe technique enjoys the advantages of simplicity, high selectivity, and sensitivity as well as convenient visual imaging with excellent spectroscopic properties (28–35). Among the reported probes, the colorimetric fluorescent probes of Cu^2+^ exhibit the potential advantages of naked-eye detection without complicated sample preparation or expensive instruments, which represent a rapid, sensitive Cu^2+^ testing method (25, 26). In addition, the development of new techniques makes it easy to quickly detect and quantify harmful levels of Cu^2+^ at low micromolarity through field tests.

To date, many colorimetric fluorescent probes consisted of large π-conjugated system such as fluorescein (36), rhodamine (37), coumarin (38, 39), anthracene (40), and BODIPY (41, 42) with obvious spectra absorption or strong fluorescence have been successfully synthesized (43). Among those probes, the fluorescein family dyes have excellent spectroscopic properties, such as long absorption and emission wavelengths, high extinction coefficients, high quantum yields, and excellent photostability, which are always introduced to construct optical sensors for metal ions (44). The sensing mechanism of these probes is based on the coordination sites to bind metal ions (45). However, the interaction between Cu^2+^ and fluorescein was rarely confirmed, which blocks our understanding of its interaction mode.

In this work, we designed and synthesized a novel fluorescent probe N4 based on a fluorescein derivative for rapid, selective, and sensitive response to Cu^2+^ in aqueous media. The fluorescent probe N4 exhibited the naked-eye detection of Cu^2+^ and a limit of detection (LOD) of 1.20 μm, indicating promise in-field applications. The solution color of N4 changes from colorless to green after the addition of Cu^2+^, with a noticeable new band at 525 nm observed under UV light. The coordination process can be detected efficiently, and the sensing mechanism is also illustrated by Job's plot, FT-IR, and mass spectra. Furthermore, biological application experiments indicated that the probes can detect Cu^2+^ in living cells, which might not only provide effective tools for Cu^2+^ imaging in biological samples, but also promote the understanding of the pathological and pharmacological effects of Cu^2+^ and its related enzymes in various diseases.

## Materials and Methods

### Materials and Reagents

Ethyl acetate, petroleum ether, ethanol, sodium hydrate, hydrochloric acid, fluorescein, and hydrazine hydrate were purchased from Tianjin Fuyu Fine Chemicals Co., Ltd (Tianjin, China). 5-Bromoindole-3-formaldehyde, copper sulfate, and dimethyl sulfoxide were purchased from Aladdin Reagent Co., Ltd (Shanghai, China). All of the reagents were of analytical grade and were utilized straight away (without further treatment). A Milli-Q system was used to create ultrapure water for all of the solutions.

### Apparatus and Instrumentation

Fluorescence analysis was carried on a HITACHI F-4500 fluorescence spectrophotometer. IR spectra were performed on a Bruker Tensor 27 spectrometer. NMR spectra were obtained on a Varian INOVA-400 MHz spectrometer (400 MHz). A Bruker micro-TOF-Q II ESI-TOF LC/MS/MS spectroscopy was used mass spectra test. Living cells imaging experiments were performed on an Olympus FV1000 confocal microscopy. Cytotoxicity analysis was recorded with the SoftMax Pro software in Spectra max190-Molecular Devices.

### Synthesis of the Probe N4

Fluorescein hydrazine was synthesized from fluorescein and hydrazine according to the literature (45). Fluorescein hydrazine (3.48 g, 10.04 mmol) and 5-bromoindole-3-carbaldehyde (1.50 g, 6.69 mmol) were dissolved in 50 ml of ethanol, refluxed for 6 h, cooled to room temperature after the reaction. The precipitate was filtered out and washed several times with absolute ethanol, and the pale yellow solid was obtained and placed in a dark place at 4°C for use; yield 43.27%, melting point 259–261°C. ^1^H NMR (400 MHz, TMS, CD_3_OD) δ 9.31 (s, 1H), 8.01 (d, J = 1.2 Hz, 1H), 7.94 (dd, J = 6.0, 1.4 Hz, 1H), 7.62 (td, J = 6.6, 1.3 Hz, 2H), 7.49 (s, 1H), 7.24-7.16 (m, 3H), 6.74 (d, J = 2.3 Hz, 2H), 6.46 (dt, J = 8.6, 5.5 Hz, 4H); ^13^C NMR (100 MHz, TMS, DMSO-d6) δ 165.9, 163.2, 158.9, 158.7, 153.2, 152.9, 152.0, 149.79, 148.9, 136.2, 133.8, 133.0, 132.8, 131.6, 129.8, 129.5, 128.9, 128.7, 128.4, 126.0, 125.6, 124.9, 124.4, 123.9, 123.2, 122.8, 114.1, 113.9, 112.6, 112.5, 112.3, 111.3, 110.4, 103.0, 102.9, 66.2, 65.1, 40.7, 40.5, 40.3, 40.1, 39.8, 39.6, 39.4, 19.0; IR (KBr, cm^−1^): 3,554, 3,402, 3,111, 1,654, 1,612, 1,503, 1,449, 1,339, 1,298, 1,265, 1,236, 1,175, 1,110, 1,078, 993, 885, 861, 792, 752, 687, 584, 529; (ESI) m/z calcd for C_29_H_18_BrN_3_O_4_ (M+Na)^+^: 574.0373. found: 574.0357.

### Cell Toxicity Study

Cell toxicity was tested by CCK-8 assay. Cells were cultured in 96-well plates and cultured at 37°C for 24 h, and then, different concentrations of probe (0.0, 2.5, 5.0, 10.0, 20.0, and 40.0 μmol/L) were added to the wells and cultured for 24 h. CCK-8 was added to each well, and the plate was incubated for another 2 h. Absorbance was measured at 450 nm. All experiments were repeated three times, and the data were presented as the percentage of control cells.

### Colorimetric Detection of Cu^2+^

The stock solution of probe N4 (1 mm) was prepared in EtOH. The solutions of biologically relevant analytes stock solutions (1 mm) were prepared in deionized water. During the titration experiments, different amounts of Cu^2+^ and 1.0 ml of 200 μm probes were mixed and filled up with phosphate-buffered saline (PBS) to 10 ml in volumetric tubes. During the interference experiments, 20 μm of Cu^2+^, 1.0 ml of N4 (200 μm), and 1.0 ml of testing species (400.0 μm) were mixed and filled up with PBS to 10 ml in volumetric tubes. During the titration experiments of ethylenediamine, 1.0 ml of 200.0 μm probes, 1.0 ml of 400.0 μm Cu^2+^, and different amounts of ethylenediamine were mixed and filled up with PBS to 10.0 ml in volumetric tubes. About 1 ml aliquots were pipetted into a 1-cm cuvette for spectral measurements. About 5 nm bandpasses were used for both excitation and emission wavelengths. For all measurements, the absorbance was recorded at 440 nm and the fluorescence intensity was recorded at 525 nm.

### Detection Limit of Probe N4

The detection limit was calculated based on the fluorescence data. To determine the δ/S ratio, the emission intensity or absorbance of N4 (20.0 μm) without Cu^2+^ was measured 10 times, and the standard deviation of the blank measurements was determined. Under the present conditions, a good linear relationship between the relative emission intensity (525 nm) and Cu^2+^ concentration could be obtained in the 0.0–30.0 μm. The detection limit is then calculated with the equation: detection limit = K × δ/S, where δ is the standard deviation of blank measurements; S is the slope between intensity vs. sample concentration. The fluorescence analysis results are as follows: linear equation: y = 49.559x-91.3 (*R*^2^= 0.9922), δ= 19.823 (*N* = 10), S = 49.559, K = 3; LOD = K × δ/S = 3 ×19.823/49.559 = 1.20 μm.

## Results and Discussion

### Spectral Studies of Probe N4 for Sensing Cu^2+^

**Figure 1:**
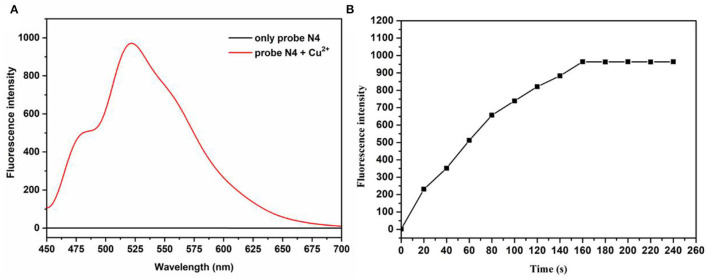
**(A)** Emission spectra of probes before and after treatment with Cu^2+^. **(B)** The time-dependent fluorescence intensity (525 nm) of probe N4 (20.0 μm) upon addition of Cu^2+^ (20.0 μm). λ_ex_ = 440 nm.

First, the optical study of the probe N4 was investigated in PBS buffer (10.0 mm, pH = 7.4)/ EtOH (1:1, v/v). As shown in the Supplementary Figure S1 and Figure 1A, when the probe was treated with Cu^2+^(20.0 μm), the fluorescence intensity at 525 nm was rapidly enhanced, which was attributed to the opening of the loop of the probe spironolactone caused by Cu^2+^. Meanwhile, the color of the probe solution changed from colorless to green under visible light, indicating that probe N4 can be used for visual detection of Cu^2+^. As shown in Figure 1B, the enhanced fluorescence intensity at 525 nm was recorded after the addition of Cu^2+^ (20.0 μm) and reached a plateau after 160 s, indicating that probe **N4** can detect Cu^2+^ rapidly.

**Figure 2:**
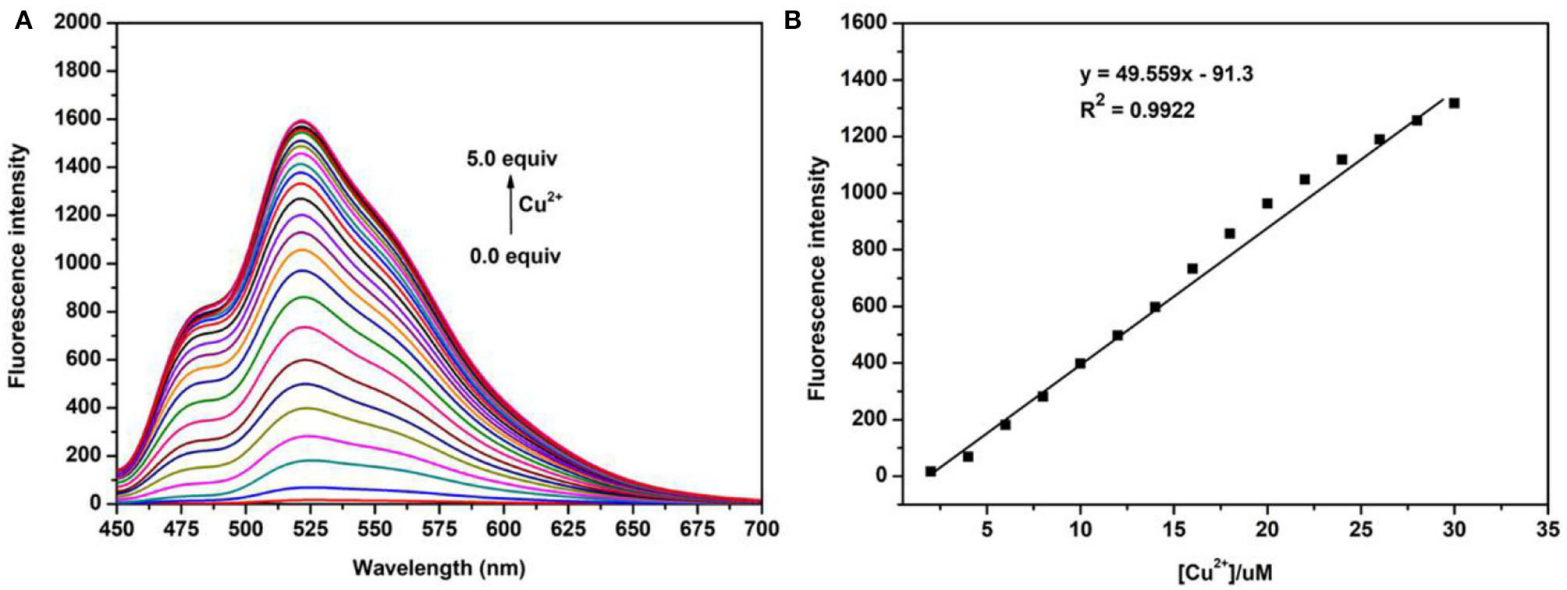
**(A)** Fluorescence titration of probe N4 (20.0 μm) upon addition of different concentration of Cu^2+^ (0.0–100.0 μm); **(B)** the linear correlation between the maximum fluorescence intensity (525 nm) and the concentration of Cu^2+^; λex = 440 nm.

Next, the titration study was carried out by adding different concentrations of Cu^2+^ (0–100.0 μm) into the solutions of the probe N4 (20.0 μm). As shown in Figure 2, the fluorescence intensity at 525 nm increased significantly with increasing Cu^2+^ concentration and reached the maximum value when the Cu^2+^ concentration up to 5.0 eq. In addition, a good linear relationship was observed between fluorescence intensity and Cu^2+^ concentration in the range of 0.0–1.5 eq., and the detection limit of probe **N4** for Cu^2+^ was calculated to be 1.2 μm. All the results showed that the probe N4 exhibited good sensitivity and the ability to quantitatively detect Cu^2+^ in related samples.

### Selectivity and Competition Studies of Probe and Effect of the pH

**Figure 3:**
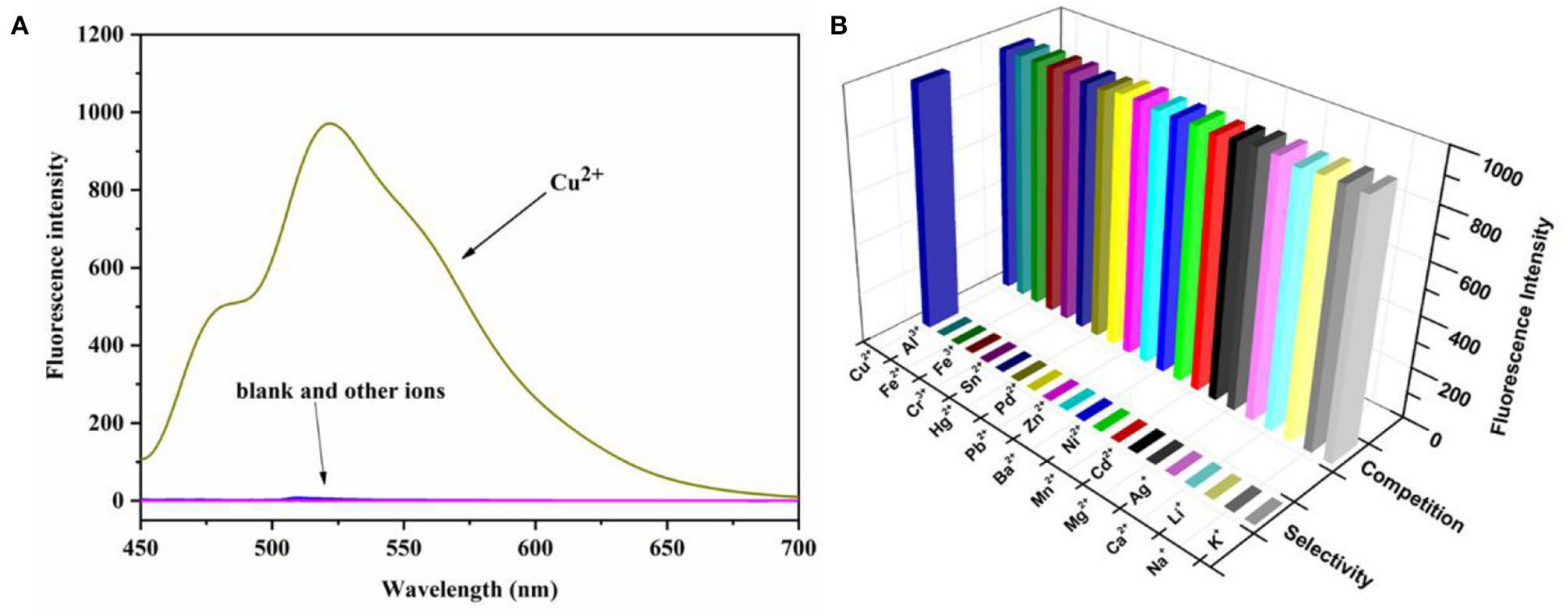
**(A)** Fluorescence spectrum of N4 (20 μm) in the presence of various metal ions K^+^, Na^+^, Li^+^, Ca^2+^, Ag^+^, Mg^2+^, Cd^2+^, Mn^2+^, Ni^2+^, Ba^2+^, Zn^2+^, Pb^2+^, Pd^2+^, Hg^2+^, Sn^4+^, Cr^3+^, Fe^3+^, Fe^2+^, Al^3+^, and Cu^2+^ (40.0 μm) in PBS buffer (10 mm, pH = 7.4)/EtOH (1:1, v/v), λ_ex_ = 440 nm. **(B)** Fluorescence intensity (525 nm) selectivity and competition of probe N4 (20 μM) in the presence of various metal ions. The pillars in the front row are: probe N4 (20.0 μM) + various metal ions. The rear pillars are: probe N4 (20.0 μM) + Cu^2+^ (40.0 μm) + various metal ions. λ_ex_ = 440 nm.

To further evaluate the selective and anti-interference ability of the probe N4 against Cu^2+^, we performed selectivity and competition studies of the probe in PBS buffer (10 mm, pH = 7.4)/ EtOH (1:1, v/v). As shown in Figure 3, with the addition of Cu^2+^, it exhibited an obvious increase in fluorescence spectroscopy at 525 nm which associated with the ring opening of the spirocyclic. In comparison, no obvious fluorescent changes were observed when other ions added. Moreover, the fluorescence properties of the probe with different ions were investigated, and the competition experiment also showed that all of the competing metal ions had no interference on the Cu^2+^-selective recognition process. In addition, the probe N4 had good selectivity for Cu^2+^ in the physiological pH range of 6.0 to 9.0 (Supplementary Figure S2).

### Proposed Mechanism

**Figure 4:**
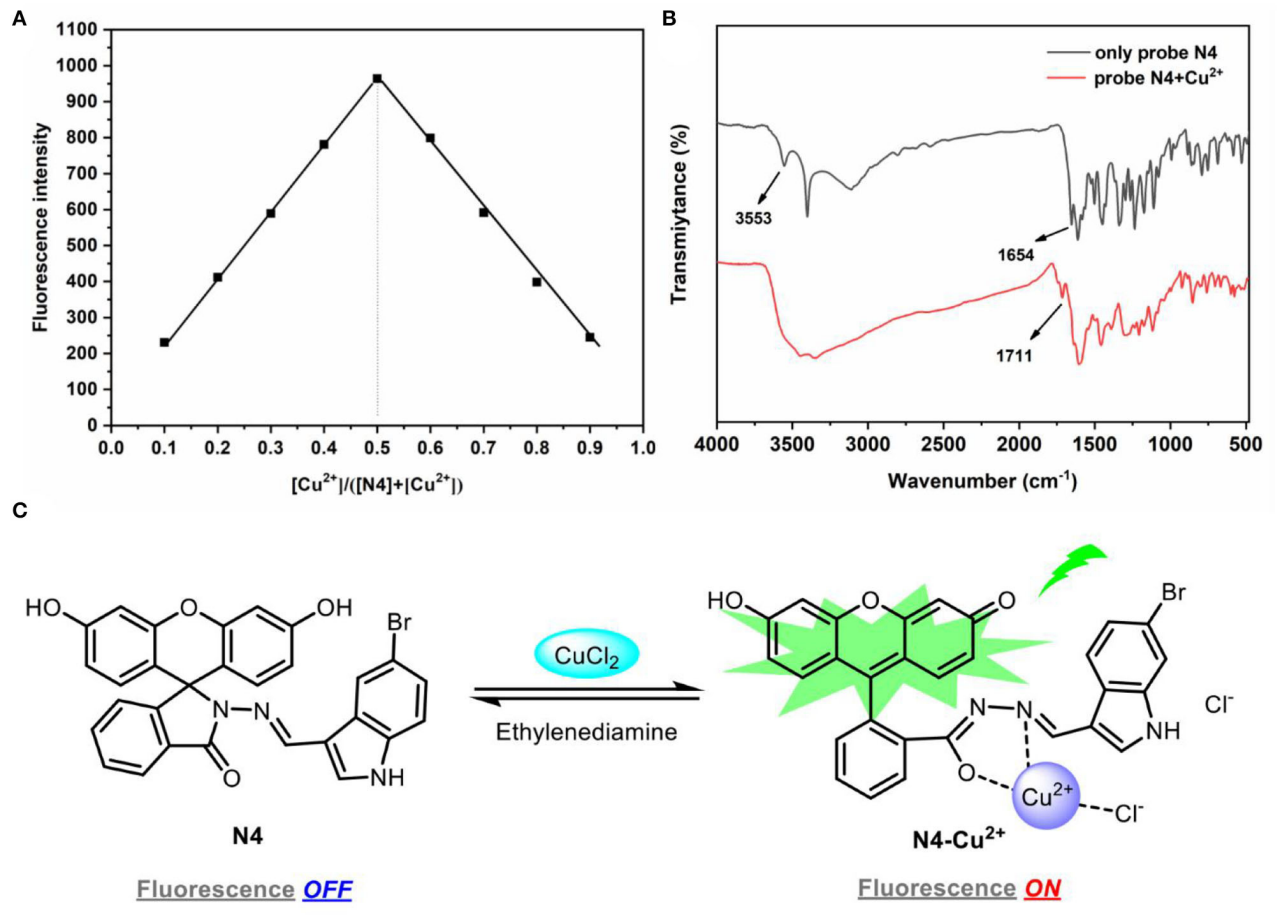
**(A)** Job's plot of probe N4 and Cu^2+^. The total concentration of probe N4 and Cu^2+^ was 40.0 μm. λex = 440 nm; **(B)** the FT-IR spectra and complex N4-Cu^2+^; **(C)** the proposed sensing mechanism of probe **N4** with Cu^2+^.

To understand the interaction between probe N4 and Cu^2+^, the mechanism was investigated by Job's plots, FT-IR, and MS analysis. The stoichiometric ratio of 1:1 between probe N4 and Cu^2+^ was gained by Job's plots (Figure 4A).

The FT-IR spectra showed that the peak change from 3,553 (–OH) to 1,711 cm^−1^ (C=O) after the reaction of probe N4 and Cu^2+^, which was attributed to the conversion of phenolic hydroxyl group to carbonyl group. The absorption peak of probe N4 at 1,654 cm^−1^ disappeared, indicating that the amide group was coordinated with Cu^2+^ (Figure 4B).

In addition, a new peak at m/z 651.1717 [C_29_H_18_BrClCuN_3_O_4_(M+CuCl)]^+^ in mass spectra was founded for the complex of probe N4 with Cu^2+^, which further illustrated the 1:1 complexation (Supplementary Figure S8). Thus, it can be supposed that the coordination of Cu^2+^ to the nitrogen atom of the Schiff base moiety and the oxygen atom of the amide carbonyl group in fluorescein as well as a free chlorine atom resulted in the Cu^2+^ induced reversible ring-opening process (Figure 4C).

### Test Strips

**Figure 5:**
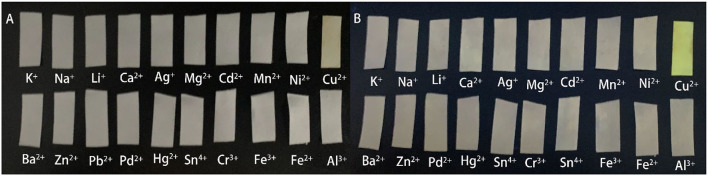
Photographs of test strips immersed in different analyte aqueous solutions under ambient light **(A)** and hand-held UV lamp at 365 nm **(B)**.

To further extend the field detection capability of the probe in real samples, we prepared probe-loaded test strips. They were subsequently immersed in different metal ion solutions (K^+^, Na^+^, Li^+^, Ca^2+^, Ag^+^, Mg^2+^, Cd^2+^, Mn^2+^, Ni^2+^, Cu^2+^, Ba^2+^, Zn^2+^, Pb^2+^, Pd^2+^, Hg^2+^, Sn^4+^, Cr^3+^, Fe^3+^, Fe^2+^, Al^3+^). It was interesting that only aqueous solutions of Cu^2+^ caused color changes that could be seen by the “naked eye” especially under UV light (Figure 5).

### Fluorescence Imaging

**Figure 6:**
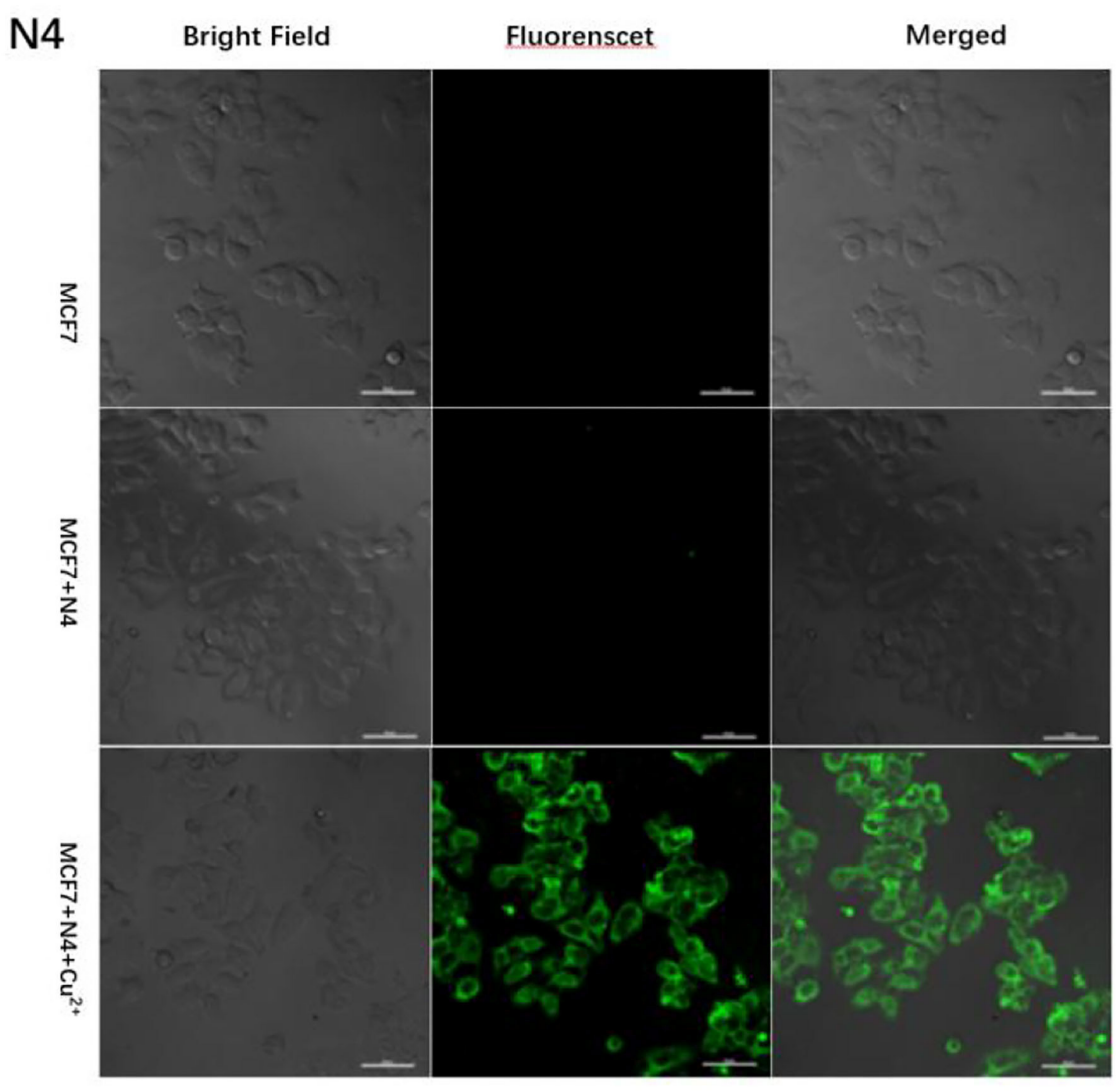
Fluorescent images of MCF-7 cells after incubation with probe N4 (40.0 μm) in the absence and the presence of Cu^2+^ (40.0 μm).

Based on the excellent performance of the probe N4, we explored the effect of probe N4 on the detection of Cu^2+^ in cell. First, the cytotoxicity of the probe to MCF-7 cells was investigated using the method of MTT. As shown Supplementary Figure S3, MCF-7 cells were incubated with different concentrations of the probe N4 (0.0–40.0 μm) for 24 h, which indicated the low cytotoxicity of the probe. To further test the bioimaging ability of probe N4 in living cells, the MCF-7 cells were cultured with the probe N4 for 30 min, and no intracellular fluorescence was observed. Then, the cells were treated with Cu^2+^ (40.0 μm) for 1 h at 37°C, and significant fluorescence from the intracellular area was found. In addition, the bright field images of cells were also seen clearly which further confirmed that the probe has good biocompatibility (Figure 6), indicating the ability of probe for tracking of Cu^2+^ in living cells.

## Conclusions

In conclusion, a novel “turn-on” fluorescent probe N4 was designed and synthesized for detecting Cu^2+^, and the probe exhibited better selectivity and sensitivity for Cu^2+^ over other ions. Meanwhile, the binding mode between probe N4 and Cu^2+^ was studied by Job's plot, FT-IR, and mass experiment, suggesting that the Cu^2+^ coordination to the Schiff base moiety and the amide carbonyl group of fluorescein induced the fluorescent emission. The probe N4 could detect Cu^2+^ in water qualitatively by test paper. More importantly, the probe was successfully used to detect Cu^2+^ in cells and was verified to have low toxicity, which presented a fantastic candidate for mapping of Cu^2+^ in related biological samples and processes.

## Data Availability Statement

The original contributions presented in the study are included in the article/Supplementary Material, further inquiries can be directed to the corresponding author/s.

## Author Contributions

XL: project administration and writing—original draft. MS: data curation and formal analysis. XJ: writing—review and editing. JC: validation and methodology. XM: formal analysis. FC and JL: supervision. BY: design the protocol and formal analysis. All authors contributed to the article and approved the submitted version.

## Funding

This work was supported by the grants from the Technology Innovation Leading Program of Shaanxi (Nos. 2020QFY07-05 and 2020TG-031).

## Conflict of Interest

The authors declare that the research was conducted in the absence of any commercial or financial relationships that could be construed as a potential conflict of interest.

## Publisher's Note

All claims expressed in this article are solely those of the authors and do not necessarily represent those of their affiliated organizations, or those of the publisher, the editors and the reviewers. Any product that may be evaluated in this article, or claim that may be made by its manufacturer, is not guaranteed or endorsed by the publisher.
