# Supplementary material for: A Highly Sensitive and Selective Fluorescein-Based Cu2+ Probe and Its Bioimaging in Cell
Source: Front Nutr. 2022 Jun 27;9:932826. doi: 10.3389/fnut.2022.932826 (PMC9271948; doi:10.3389/fnut.2022.932826)
Supplement: Supplementary file 1 [file Presentation_1.pdf]

A Highly Sensitive and Selective Fluorescein-Based Cu^{2+} probe and Its Bioimaging in Cell

CONTENTS

1. Experimental

2. Spectroscopic properties of probe **N4**

3. MTT Assay

4. ^{1}H NMR, ^{13}C NMR and MS spectra

1. Experimental

1.1 Synthesis of probe N4

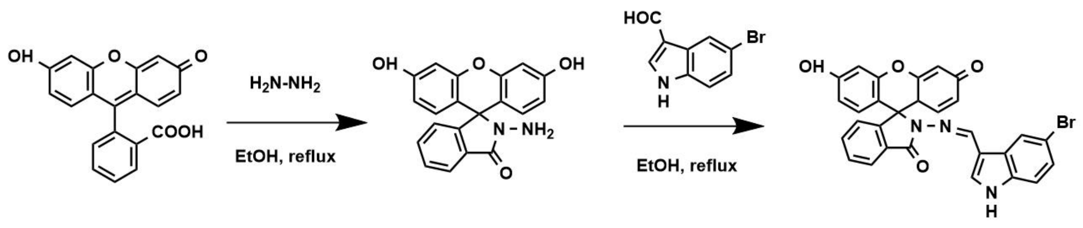

Scheme S1 Synthesis route of probe **N4**

1.2 Cell culture experiments

MCF-7 cells were obtained from the Laboratory Center of Shaanxi Province People's Hospital. MCF-7 cells were grown on glass-bottom culture dishes using Dulbecco's Modified Eagle Medium (DMEM) supplemented with 10% (V/V) fetal bovine serum (FBS) and 50 μg/mL penicillin-streptomycin at 37 ℃ in the humidified atmosphere with 5% CO_2 and 95% air. The growth medium was then removed and washed three times with FBS. The cells were pretreated with 20.0 μM probe **N4** for 30 min at 37 ℃, washed with PBS (pH 7.4) twice and imaged. Then the cells were incubated with 20 μM $CuCl_2$ for 30 min at 37 ℃, washed with PBS (pH 7.4) twice and imaged.

2. Spectroscopic properties of probe N4

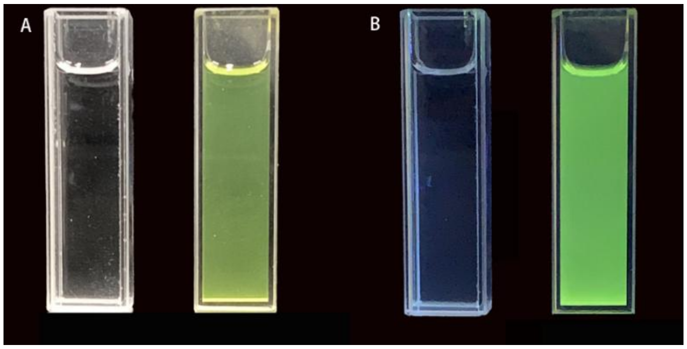

FIGURE S1. Photos of the probe **N4** in the absence and the presence of Cu^{2+} under fluorescent lamp and ultraviolet lamp

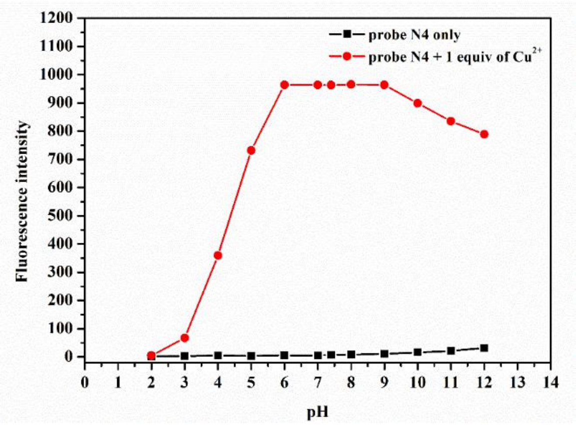

FIGURE S2. Fluorescence responses of probe **N4** (20 μM) in the absence and presence of Cu^{2+} (20.0 μM) with different pH conditions (λex = 440 nm).

3 MTT Assay

MCF-7 cells were seed in 96-well plates at density of 5000 cells/well. Plates were maintained at 37 °C in a 5% CO_2/ 95% air incubator for 24 hours. Then the culture medium in each well were replaced by fresh medium containing different concentrations of probe **N4**. After 24 hours treatment, into each well, 15.0 μL MTT solutions (5.0 mg/mL in phosphate buffer solution) were added. After 4 hours incubation at 37 °C, the absorbance of each well at 440 nm was recorded by the Elx800 Absorbance Microplate Reader.

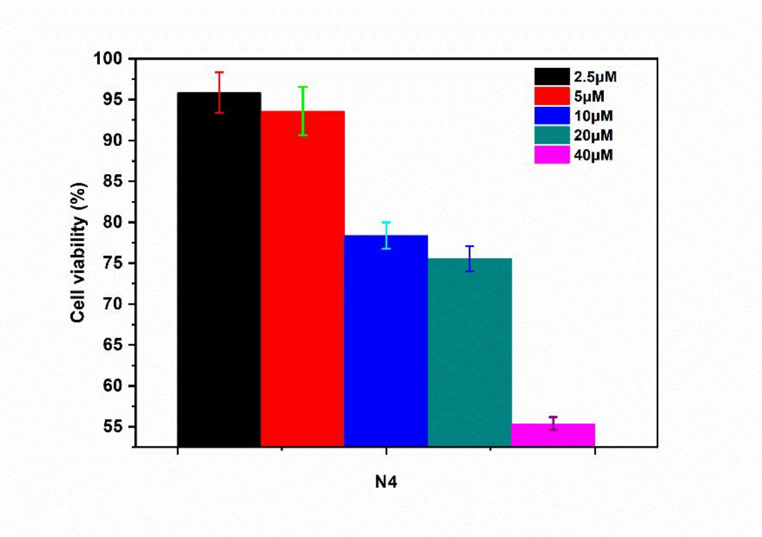

FIGURE S3. MTT assay of MCF-7 cells in the presence of different concentrations of probe **N4** (2.5μM; 5.0 μM; 10.0 μM; 20.0 μM; 40.0 μM).

4 ^{1}H NMR, ^{13}C NMR and MS spectra

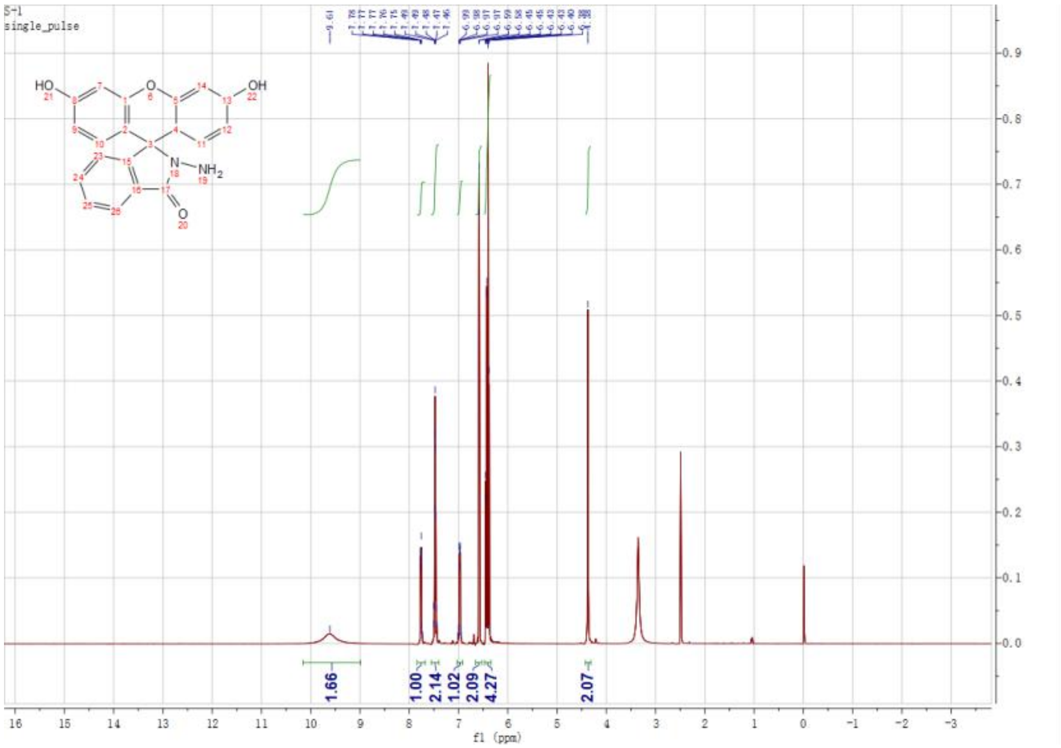

FIGURE S4. ^{1}H NMR spectrum of fluorescein hydrazine in DMSO.

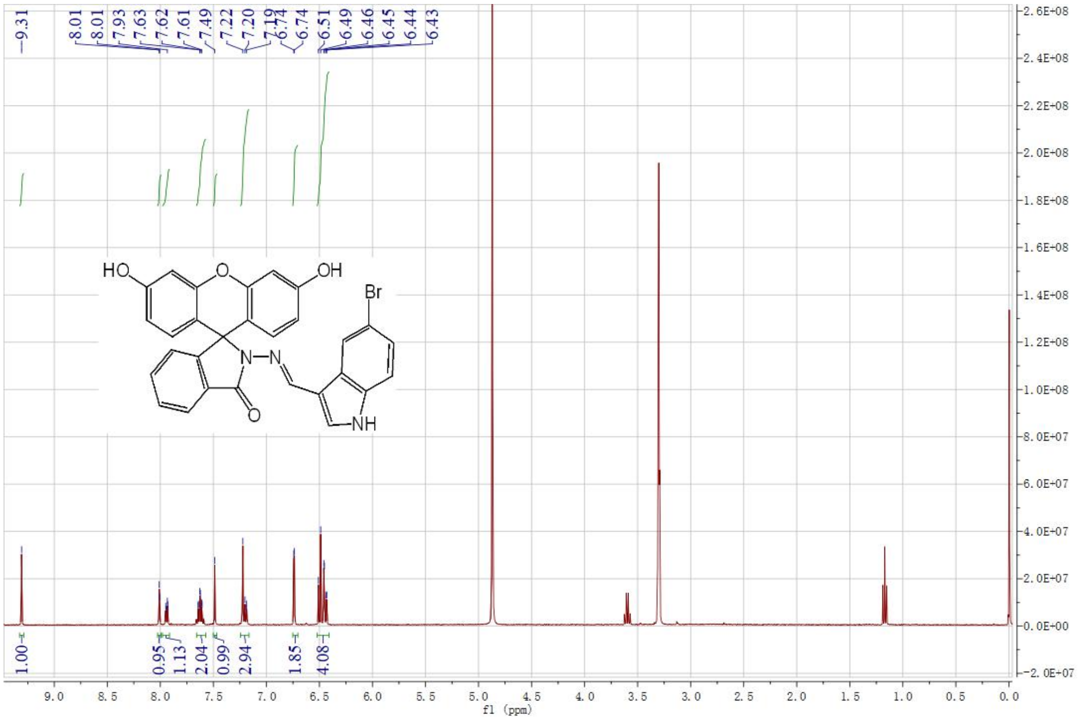

FIGURE S5. ^{1}H NMR spectrum of probe **N4** in $CDCl_3$.

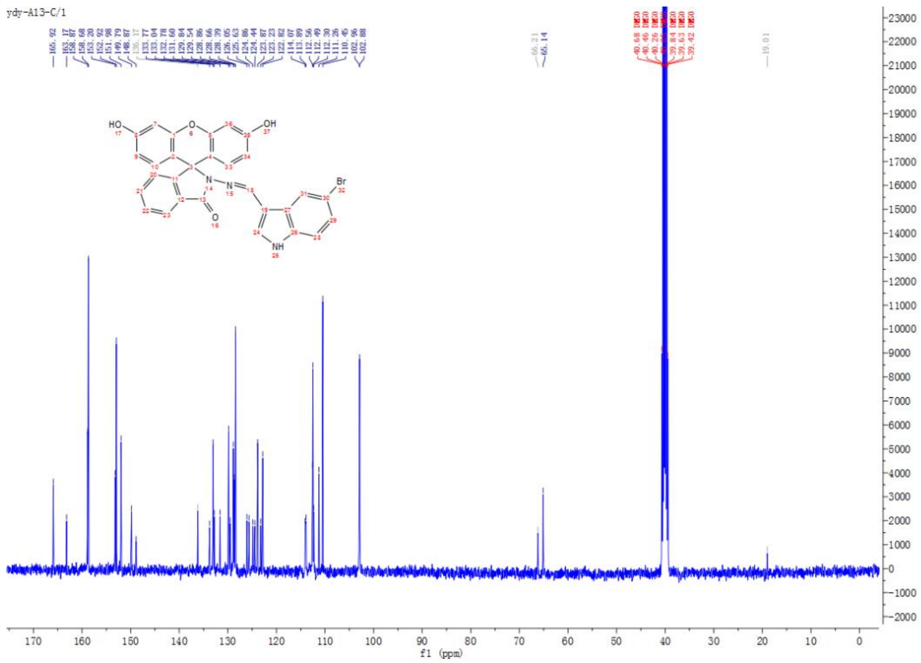

FIGURE S6. ^{13}C NMR spectrum of probe **N4** in DMSO.

Mass Spectrum SmartFormula Report

Analysis Info

Analysis Name	C:\Users\ÂíÑ©ºÆ\Desktop\ÂÛÎÄ20220425\µÚÈýÕÂÊý¾Ý´¦Àí£-N4£-20190820\4£-ÖÊÆ×Êý¾Ý\ybq_lengxin_A13	Acquisition Date	2018/5/3 15:00:22
Method	tune_low_200-600.m	Operator	service
Sample Name		Instrument / Ser#	micrOTOF-Q II 10280
Comment			

Acquisition Parameter

Source Type	ESI	Ion Polarity	Positive	Set Nebulizer	0.4 Bar
Focus	Not active	Set Capillary	4500 V	Set Dry Heater	180 °C
Scan Begin	50 m/z	Set End Plate Offset	-500 V	Set Dry Gas	4.0 l/min
Scan End	3000 m/z	Set Collision Cell RF	110.0 Vpp	Set Divert Valve	Source

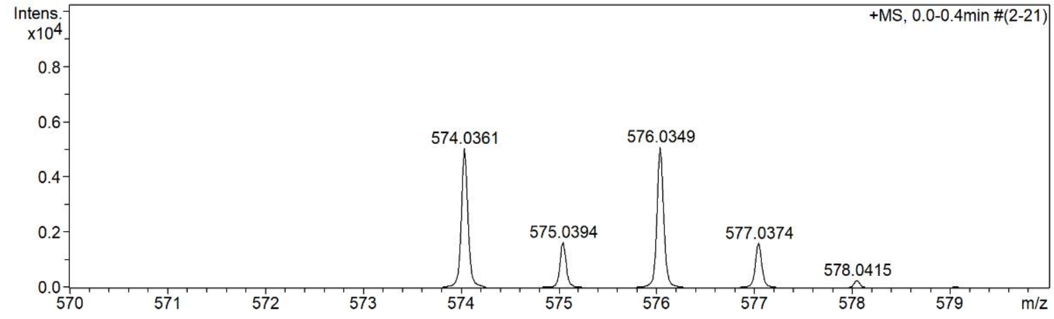

Meas. m/z	#	Formula	Score	m/z	err [mDa]	err [ppm]	mSigma	rdb	e¡¥Conf	N-Rule

FIGURE S7. Mass spectrum of probe **N4**.

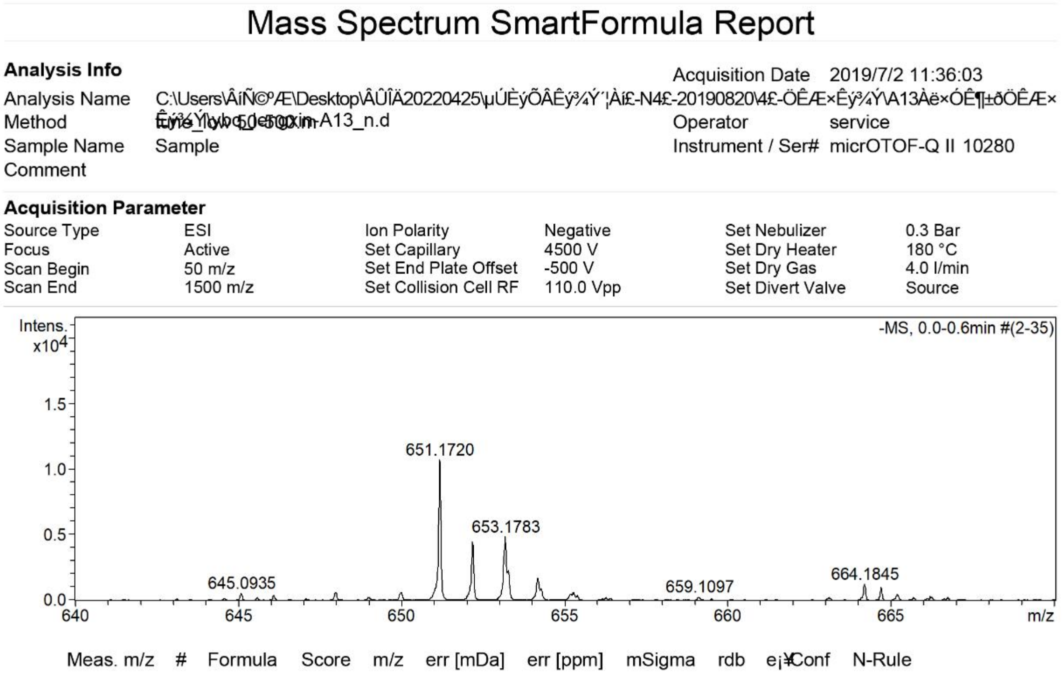

FIGURE S8. Mass spectrum of complex **N4-Cu.**
